# The impact of cancer therapy on cognition in the elderly

**DOI:** 10.3389/fphar.2013.00048

**Published:** 2013-04-19

**Authors:** Victoria Mandilaras, Doreen Wan-Chow-Wah, Johanne Monette, Francine Gaba, Michèle Monette, Linda Alfonso

**Affiliations:** ^1^Division of Geriatric Medicine, Jewish General HospitalMontreal, QC, Canada; ^2^Internal Medicine Residency Training Program, McGill UniversityMontreal, QC, Canada; ^3^Segal Cancer Centre, Jewish General HospitalMontreal, QC, Canada; ^4^Solidage – McGill University – Université de Montréal Research Group on Frailty and Aging, Centre for Clinical Epidemiology and Community Studies, Jewish General HospitalMontreal, QC, Canada

**Keywords:** cognition, chemotherapy, chemobrain, geriatric oncology, elderly, cancer

## Abstract

Cancer and cancer therapy-related cognitive impairment (formerly known as chemobrain or chemo-fog) are often described in the literature. In the past, studies have failed to prove the existence of cancer therapy-related cognitive dysfunction. However, more recently, prospective trials have shown that patients undergoing chemotherapy do display impairment in specific cognitive domains. Aging confers an increased risk of developing cancer, as well as cognitive impairment. The Geriatric Oncology clinic of the Segal Cancer Centre, Jewish General Hospital in Montreal was founded in 2006 to address the unique needs of older cancer patients. We will describe two cases of cancer therapy-related cognitive impairment from our Geriatric Oncology clinic. The first case is that of a 75 year old male diagnosed with stage III non-small cell lung carcinoma who complained of forgetfulness since starting carboplatin-paclitaxel. The second case is that of a 65 year old female diagnosed with stage I, estrogen-receptor-positive breast cancer who had undergone lumpectomy followed by adjuvant cyclophosphamide, methotrexate and fluorouracil chemotherapy, radiation therapy and was on exemestane when she was evaluated. We will also briefly review the literature of cancer therapy-related cognitive impairment.

## INTRODUCTION

Cancer treatment is a field that has made great advances in the last century. Survival has been greatly affected by novel therapies. However, with increased long-term survival from cancer, the long-term effects of treatment remain to be seen. The notion of the effects of chemotherapy on cognition, although initially described in the 1970s, only began to garner attention in the late 1990s as it became increasingly recognized as a common and significant symptom in cancer survivors (Hede, 2008). Aging is not only the most important risk factor for developing cancer but also a major predisposing factor to cognitive dysfunction. Forty-three percent of new cancer cases occur in those aged 70 and above, and 60% of cancer deaths occur in this age group (Canadian Cancer Society’s Steering Committee on Cancer Statistics, 2012). Similarly, the number of Canadians living with cognitive impairment, including dementia, now stands at 747,000 and will double to 1.4 million by 2031 (Alzheimer Society of Canada: “A new way of looking at the impact of dementia in Canada”, September 2012). Older cancer patients with pre-existing cognitive impairment are at greater risk of worsening cognitive dysfunction during cancer treatments, which can subsequently lead to functional decline and loss of autonomy (Smith et al., 2009). The added insult that chemotherapy may incur can impact these patients to a greater extent than their younger counterparts. Cognitively impaired individuals may also not have the capacity to fully comprehend their cancer diagnosis and proposed treatments, thus rendering informed decision-making challenging.

Researchers are now aiming to study cognitive impairment due to cancer treatment more systematically in the elderly. Hurria et al. (2006) were among the first to investigate cognitive decline in patients over the age of 65 receiving chemotherapy. Thirty-one patients with breast cancer receiving adjuvant chemotherapy underwent neuropsychological testing prior to and after treatment. It was observed that 39% of patients experienced a decline in cognitive domains including visual memory, spatial function, psychomotor function, and attention. The team of (Kvale et al., 2010) also attempted to show the effect of cancer treatment in the geriatric population. Their study used data from a previous trial (ACTIVE trial) looking at the effect of cognitive training on 2,802 individuals aged 65–94 years. They identified subjects who developed cancer during their enrolment in the study who had a baseline assessment at least 2 years prior to their diagnosis. Thirty-seven of these patients had a cancer diagnosis and were assessed after treatment and followed with annual assessments. They were compared to age-matched control groups without a cancer diagnosis. A trend toward cognitive decline was found after treatment for cancer although it was not statistically significant (*p* = 0.07). Poor baseline cognitive functioning, older age and increased number of depressive symptoms were predictive of poorer post-therapy cognitive test scoring (*p* = 0.047). Another study by Minisini et al. (2008) prospectively looked at a group of 61 patients with a diagnosis of cancer. These patients were divided into three groups: those who did not receive cancer treatment, those who received chemotherapy, and those who received hormonal therapy. Cognitive assessments were conducted at the first visit and at three follow-up visits for a duration of 6 months. The mean ages were 71, 71.5, and 72 years old in the group without cancer treatment, with chemotherapy, and on hormonal therapy, respectively. Interestingly, there was no significant decline in cognitive testing scores in the treatment groups after treatment at the 6 month evaluation. However, when each cognitive domain was analyzed, the treatment groups showed a decline in attention capacity, learning memory and total memory. The authors did not comment on statistical significance of these results. In addition, the patients were only evaluated 6 months after therapy which may be too soon to properly assess the impact of cancer treatment on cognition. It is also important to note that all these studies are limited by their small sample size.

## BACKGROUND

The Geriatric Oncology clinic of the Segal Cancer Centre, Jewish General Hospital, a McGill University teaching hospital in Montreal, was founded in 2006 to address the unique needs of older cancer patients. Patients are referred to our clinic for opinion on cancer treatment plan, assessment of cognitive impairment before, during or after cancer therapy, management of multiple comorbidities, and functional decline. All patients undergo a comprehensive geriatric assessment (CGA) which is comprised of a multidimensional evaluation looking at health and functional status, cognition, mood, nutrition, mobility, and frailty markers. Specifically, our standard cognitive assessment includes administering the mini-mental state examination (MMSE; Folstein et al., 1975) and the Montreal Cognitive Assessment (MoCA; Nasreddine et al., 2005). The purpose of using the MoCA is to detect mild cognitive impairment in patients who score well on the MMSE (i.e., a score> 26/30). Certain patients whose MMSE and MoCA scores are inconclusive are referred for further evaluation by our Neuropsychologist. Finally, ancillary tests such as routine blood tests [including thyroid-stimulating hormone (TSH), Calcium level, vitamin B12, and folate levels] and brain imaging [computed tomography (CT) scan, magnetic resonance imaging (MRI), or positron emission tomography (PET) scan] are performed to complete the work-up. Thus far, we have prospectively gathered data from a total of 270 cancer patients. Of these, 212 patients were found to have cognitive impairment based on an MMSE and/or MoCA score less than 26/30. The majority of patients (68%) were female and the mean age was 79 years old (SD = 8.1). The majority (68%) had no post-secondary education. We further subdivided the patients into three diagnostic categories: mild cognitive impairment *n* = 72 (27%), dementia *n* = 54 (20%), possible/probable cancer and/or cancer therapy-related cognitive impairment *n* = 44 (16%). Below, we present two cases from our clinic whereby cancer treatment may have impacted on the patient’s cognitive function. Of those whom we thought may have cancer therapy-related cognitive impairment, we chose to present patients who showed improvement on cognitive testing after cessation of chemotherapy. These cases highlight the inherent challenges in establishing a diagnosis of cancer therapy-related cognitive impairment in older patients.

### CASE 1

The first case is a 75 year old male previously known only for bilateral cataracts. He was diagnosed with stage III non-small cell lung carcinoma at the age of 74 years following investigations for a persistent cold. A routine X-ray revealed a right lower lobe lung mass which was diagnosed as non-small cell lung cancer (NSCLC) following a CT chest and bronchoscopy. He began gemcitabine but experienced an adverse reaction and it was changed to carboplatin and paclitaxel.

Previous to his cancer diagnosis and treatment, he lived in an autonomous seniors’ residence with his wife. He was retired but had previously worked as a laboratory technician and had a high school level education. He was independent in all his activities of daily living (ADLs) and most instrumental ADLs (IADLs) except for cooking and shopping which were always carried out by his wife. He ambulated independently. His wife noted that he was becoming more forgetful in the 4 years prior to his cancer diagnosis. However, once initiating carboplatin/paclitaxel the patient himself began complaining of forgetfulness and was referred to our clinic by his pulmonary oncologist. Cognitive testing showed a MMSE of 27/30 and a MoCA of 21/30 (normal score is ≥ 26). He denied any depressive symptoms and scored 2/15 on the geriatric depression scale (GDS) which is negative for depression. A CT head was significant only for an old lacunar infarct in the lentiform nucleus. A PET scan did not reveal any metastatic disease.

A complete work-up was performed at the first visit and all blood tests were normal. A follow-up visit 5 months later while he was still receiving chemotherapy revealed cognitive testing scores of 30/30 on the MMSE and 23/30 on the MoCA test.

Two months after the cessation of the chemotherapy, the patient’s cognitive testing scores improved, with a MMSE of 30/30 and a MoCA of 27/30). The patient also reported that, subjectively, he found his memory to be improved.

It is important to note that the patient had a history of memory problems predating the cancer diagnosis and treatment by 4 years. In fact, it is possible that the patient may have been suffering from baseline mild cognitive impairment. This may have put him at higher risk of delirium secondary to chemotherapy. Furthermore, the improvement of his cognitive testing scores could be explained by a delirium which resolved given the relatively quick resolution of his cognitive deficits after cessation of chemotherapy.

### CASE 2

The second case is a 65 year old female diagnosed with stage I, estrogen receptor (ER)/progesterone receptor (PR) positive breast cancer 3 years prior to our evaluation in the clinic. She had undergone segmental mastectomy followed by adjuvant cyclophosphamide, methotrexate and fluorouracil (CMF) chemotherapy, radiation therapy and was on exemestane at the time of evaluation. She had been complaining of decreased memory and attention span since starting chemotherapy. The patient underwent a CGA. She scored 30/30 on the MMSE and 21/30 on the MoCA during her initial visit. Her depression screening test was negative (GDS 3/15) and she denied depressive symptoms. Again, a routine work-up for common causes of her cognitive impairment was negative (TSH, vitamin B12 level, CT head were all normal). Neuropsychological testing revealed that her cognitive profile was mostly normal but that she did display attention and concentration deficits. In particular, she had deficits in executive functioning which indicated weak frontal lobe function and was diagnosed with mild cognitive impairment.

The patient returned to clinic for an evaluation 1 year later. At that time, she scored a 27/30 on the MoCA which was improved from previous. The patient noted that her memory was better as well. She was still on exemestane at the time of her second visit. Again, these results illustrate how the exact mechanism for the decline and subsequent improvement are difficult to elucidate. Her cognitive impairment was present years after her chemotherapy and improved within 1 year of coming to our attention. Furthermore, the patient’s symptoms cannot be explained by her use of hormone therapy as she improved while still using exemestane.

## DISCUSSION

The evidence remains conflicting with regards to cancer therapy-related cognitive dysfunction. Certain cross-sectional and prospective studies have failed to prove its existence (Ruzich et al., 2007; Du et al., 2010) while other prospective trials using in-depth neuropsychiatric testing have shown that cancer therapy does cause impairment in specific cognitive domains (Falleti et al., 2005; Stewart et al., 2006, 2008; Quesnel et al., 2008; Jansen et al., 2011). To explain these results, we turned to the literature and below we present a brief summary of the research surrounding this issue. It has widely been accepted that patients who have been diagnosed with cancer may experience cognitive difficulties. Whether this effect is due to cancer therapy, however, is still debated as confounding factors such as the cancer itself and its effect on mood come into effect (Schilder et al., 2010).

Recent studies have suggested that the prevalence of cancer therapy-related cognitive dysfunction ranges from 15 to 60% (Ahles and Saykin, 2007); (Wefel et al., 2010). The lack of pre-treatment screening makes it impossible to know to what extent cognitive derangements can be attributed to therapy alone. Furthermore, the lack of consensus on what kind of neuropsychological testing to use makes it difficult to be able to corroborate results of various studies.

Several prospective trials that have patients undergo neuropsychological testing prior to chemotherapy have shown that there is some degree of cognitive deficits in a subset of cancer patients at baseline that cannot be explained by psychiatric symptoms (Hermelink et al., 2007; Hess et al., 2010; Jansen et al., 2011). Even more interesting is that both Jansen et al. (2011) and Hermelink et al. (2007) have shown that some cancer patients improve cognitively after chemotherapy while others deteriorate. One study aiming to explain this result looked at cancer patients treated with and without chemotherapy and compared them with cardiac patients and healthy controls (Mehlsen et al., 2009). Of note, the healthy controls were participants recruited through the test groups, colleagues, and students. In all, 34 cancer patients, 12 cardiac patients and 12 healthy controls were recruited. The age of participants ranged from 18 to 65 years old. No significant change in neuropsychological testing between the three groups was observed at the various time points of testing. It was concluded that chemotherapy does not confer an increased risk for cognitive dysfunction nor does it protect against it. Limits of this study include the small sample size, the discrepancy in follow-up between the cancer patients (6 months) and the controls (3 months) and the wide age range which may skew the results of cognitive performance. Immediate cognitive deficits may have been missed as the cancer patients were evaluated too long after chemotherapy.

While many studies have demonstrated a negative effect of chemotherapy on cognition immediately after therapy, the long-term effects remain to be seen. One study demonstrated that breast cancer survivors over the age of 65 years who received chemotherapy had evidence of neurocognitive deficits 10 years after chemotherapy compared to matched controls (Yamada et al., 2010). These patients actually did significantly worse on dementia screening (MMSE 27.6 vs. 29.3 with *p* < 0.001). Furthermore, the cancer survivors scored significantly worse on domains of attention, working memory, psychomotor speed, and elements of executive functioning on neuropsychological testing. Similarly, another study looked at cognitive function in breast cancer survivors 20 years after their diagnosis (Koppelmans et al., 2012). In total, 196 patients who underwent CMF chemotherapy during the years 1976–1995 were recruited. They underwent neuropsychological testing and were compared to controls who had never had cancer. This study was able to demonstrate that, even 20 years after the completion of chemotherapy, treated patients still performed worse on learning, verbal memory, information processing, inhibition, and psychomotor speed. It is interesting to note that the chemotherapy group performed no differently on dementia screening than did the random controls.

Other studies in breast cancer patients have managed to show some reversibility after the cessation of chemotherapy (Jansen et al., 2011). One study actually observed that patients generally did better on neuropsychological testing as time elapsed since the time of their chemotherapy. However, 21% of the group still displayed deficits on neuropsychological testing on average 9 months post-treatment, chiefly in verbal-semantic memory (Weis et al., 2009). Similarly, a study in 14 stage III NSCLC patients treated with cisplatin/etoposide and radiotherapy showed similar results. Of note, the investigators found that 71% of patients displayed cognitive dysfunction prior to any treatment. Dysfunction persisted 1 month after treatment but started to dissipate as early as 7 months. The authors attribute this to the recovery from neurotoxicity of chemotherapy (Whitney et al., 2008).

## CONCLUSION

Cognitive dysfunction associated with cancer treatment, particularly chemotherapy and hormonal therapy, is a complex phenomenon. Various studies show either inconclusive or, at times, contradictory findings as it is difficult to dissect the effect of cancer itself, its effect on mood and energy and its treatment on cognitive function. The assessment of cognitive function, especially before cancer treatments, as proposed by both the SIOG (International Society of Geriatric Oncology) and the senior adult oncology guidelines of the NCCN (National Comprehensive Cancer Network) is of particular importance in older cancer patients. Screening for cognitive deficits at baseline provides valuable information to determine patients’ ability to understand their cancer diagnosis and proposed treatments as well as their decision-making capacity. It may also impact on the decision of whether a patient is a candidate for treatment.

As demonstrated in the first case study, a diagnosis of cognitive impairment at baseline confers a higher risk of delirium at any time during a patient’s cancer care trajectory. Thus, recognizing the possibility of delirium as a diagnosis and detecting problems earlier can allow for preventative strategies to minimize the risk of delirium. Another common problem in the elderly which can impact on cognition is polypharmacy, often in the context of multiple chronic diseases. Particular attention should be placed on avoiding the use of inappropriate drugs in older adults (American Geriatrics Society 2012 Beers Criteria Update Expert Panel, 2012) and minimizing drug–drug and drug–disease interactions. Clinicians caring for the increasing number of older adults with cancer should appreciate the importance and relevance of cognitive function in that population. Moreover, the potential deleterious impact of cancer therapy on cognition adds another level of complexity which should be acknowledged and addressed. Further research in this field is needed and the advent of more prospective studies conducted over a longer time period will hopefully shed more light on this pertinent topic.

## Conflict of Interest Statement

The authors declare that the research was conducted in the absence of any commercial or financial relationships that could be construed as a potential conflict of interest.
